# Second Mesiobuccal Canal Evaluation Features with Cone-Beam Computed Tomography

**DOI:** 10.1155/2019/5856405

**Published:** 2019-04-09

**Authors:** Oleg Mordanov, Zurab Khabadze, Fatima Daurova, Inna Bagdasarova, Andrei Zoryan, Alena Kulikova, Anastasiya Blokhina, Rita Mustafaeva, Yusup Bakaev, Saida Abdulkerimova

**Affiliations:** Department of Therapeutic Dentistry, RUDN University, Medical Institute, Moscow, Russia

## Abstract

**Aim:**

The aim of the study is to evaluate the difference in MB2 prevalence with different slice thicknesses in maxillary first molars.

**Materials and Methods:**

Two hundred nonfilled MB2 canals in maxillary first molars of 156 people (75 females and 81 males) aged from 20 to 73 years old were evaluated with CBCT with different slice thicknesses: 0.5 mm, 1 mm, 3 mm, and 10 mm. A general analysis was performed out, as well as in the age groups and on gender groups.

**Results:**

Visualization with 0.5 mm and 1 mm slice thicknesses was 100% and generally equal, in both the male and the female group. General MB2 visualization with 3 mm slice thickness was 42% and 29% for the male group and 27% for the female group. No canals were visualized with 10 mm slice thickness. The study did not demonstrate a statistical difference in the MB2 prevalence between gender and age groups with the 3 mm slice thickness.

**Conclusion:**

The most valuable way to evaluate the root canal system in first maxillary molars with CBCT is using 1 mm slice thickness for both genders and every age group.

## 1. Introduction

An understanding of the root canal morphology significantly reduces difficult challenges while preparing access to the cavity as well as during cleaning, shaping, and filling procedures [1, 2]. Inadequate knowledge concerning the anatomy of the root canal is a major cause of treatment failure [3].

If apical periodontitis reoccurs after root canal treatment, it is considered as a persistent lesion, usually attributed to endodontic treatment failure [4]. Endodontic failure in the maxillary first molars can be caused by the inability to detect a second mesiobuccal (MB2) canal [5].

In an attempt to facilitate location of accessory canals such as the MB2 and to reduce treatment failure rates, cone-beam computed tomography (CBCT) has been introduced into endodontic practices [6, 7]. It provides 3D images of the tooth structure with no destruction and enables a thorough assessment of the internal and external morphology of the root canal system [7, 8]. CBCT scans can better visualize MB2 canals compared to other modalities such as digital radiography [9].

Some studies showed the improvement in different anatomical structures visibility with the change of slice thickness on CBCT scans [10, 11]. Furthermore, different artifacts reduce diagnostic ability as a result of the materials used such as gutta-percha [12].

Thus, the aim of the study is to evaluate the difference in MB2 prevalence with different slice thicknesses in maxillary first molars.

## 2. Materials and Methods

**Figure 1:**
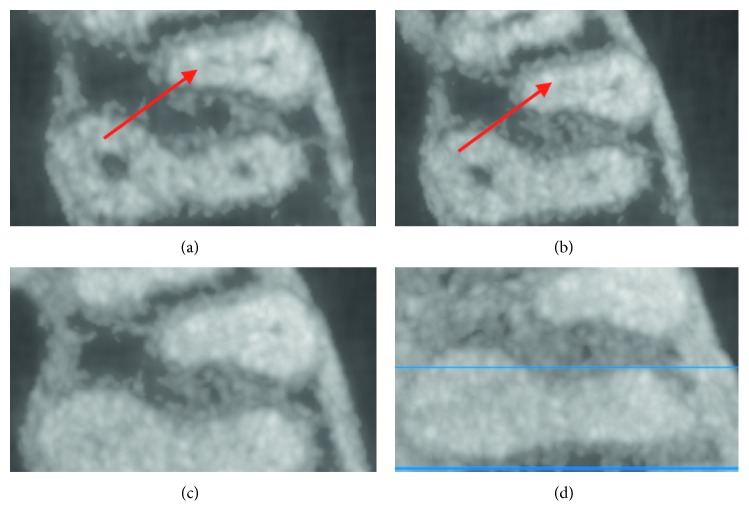
Roots of the maxillary first molar. The same tooth and slice can be seen in every picture. The red arrow shows MB2. (a) 0.5 mm slice thickness. (b) 1 mm slice thickness. (c) 3 mm slice thickness. MB2 looks obliterated, and it is not visualized. (d) 10 mm slice thickness. None of the canals is visualized.

Two hundred nonfilled MB2 canals in maxillary first molars were evaluated using Ez3D (Vatech) software with different slice thicknesses: 0.5 mm, 1 mm, 3 mm, and 10 mm (Figure 1). The study included the teeth of 156 people (75 females and 81 males) aged from 20 to 73 years old. Written consent was signed by all individuals before taking the procedure.

The patients with trauma, with bone disorders, undergoing bisphosphonate therapy, with anamnesis of surgical procedures, and with pathological disorders of the anterior maxilla were excluded from the study.

All CBCT scans were made with the CBCT device with the following characteristics: 0.2 mm/0.3 mm voxel size; 0.5 mm focal spot; 18 sec scanning time; 55–99 kB/4–16 mA tube voltage.

A general analysis was performed out, as well as in the age groups (18–39; 40–59; and 60 and more years old) and on gender groups. The one-way ANOVA test was provided with StatPlus 6 (AnalystSoft). Age and gender groups were analyzed within slice thickness groups.

## 3. Results

**Figure 2:**
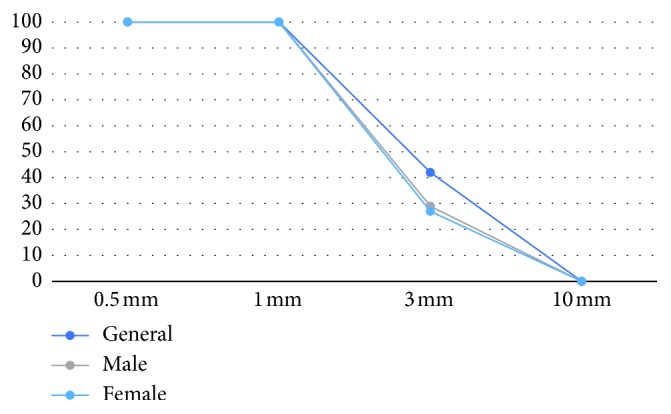
Total and gender decrease of MB2 prevalence with the increase of slice thickness in the same group of the root.

This CBCT study of 200 MB2 canals revealed a decrease in MB2 canals prevalence with an increase in slice thickness (Figure 2). Visualization with 0.5 mm and 1 mm slice thicknesses was 100% and generally equal (*n*=200), in both the male (*n*=96) and the female (*n*=104) group. General MB2 visualization with 3 mm slice thickness was 42% (*n*=84) and 29% (*n*=28) for the male group and 27% (*n*=56) for the female group. No canals were visualized with 10 mm slice thickness.

The MB2 prevalence between the 0.5 mm and 1 mm slice thicknesses was equal; in general, the difference between the 0.5 mm/1 mm slice thickness and the 3 mm slice thickness (42%, *n*=84) was statistically significant (*p* < 0.01). The same significant difference between the 0.5 mm/1 mm slice thickness and the 3 mm slice thicknesses is within the male and female groups (*p* < 0.01).

**Figure 3:**
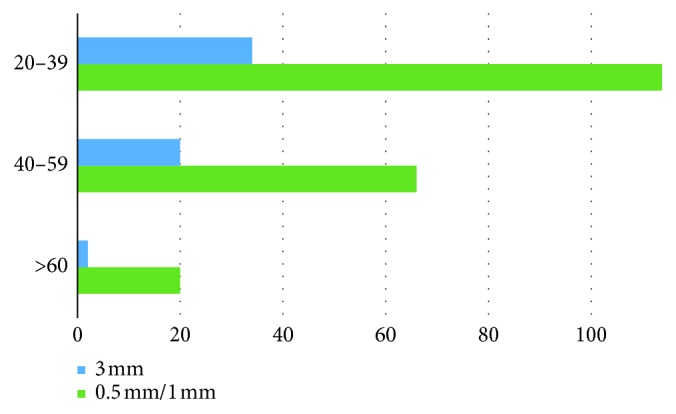
Total MB2 prevalence in the age groups according to slice thickness.

**Figure 4:**
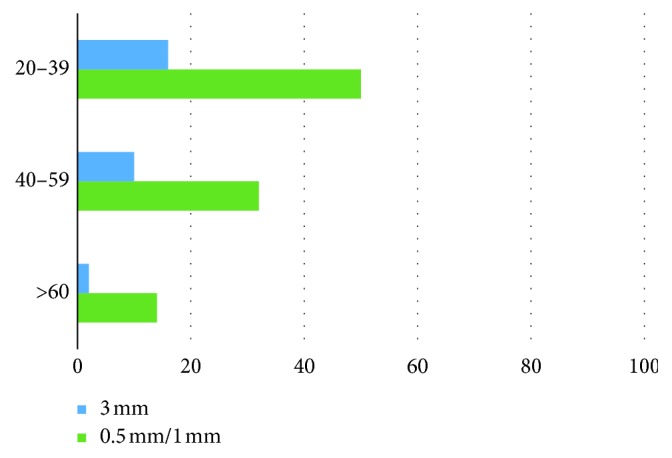
MB2 prevalence in the male age groups according to slice thickness.

**Figure 5:**
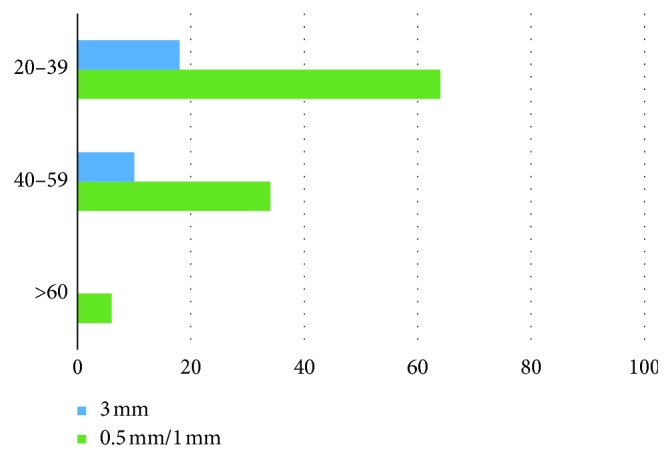
MB2 prevalence in the female age groups according to slice thickness.

All patients were divided in 3 age groups. The first group (20–39 years) involved the teeth of 50 males and 64 females; the second age group (40–59 years) involved the teeth of 32 males and 34 females; and the third group involved the teeth of 14 males and 6 females. The difference in visualization among age groups is shown generally (Figure 3), for males (Figure 4) and for females (Figure 5).

The decrease in total visualization was also statistically significant among every age group (*p* < 0.01 consequently). Both males and females have the significant decrease of MB2 visualization due to change in the slice thickness from 0.5 mm/1 mm to 3 mm (*p* < 0.01 for both).

However, the total difference between the 20–39 age group and the 40–59 age group using the 3 mm slice thickness is not statistically significant (*p*=0.6) and neither is it for the 20–39 age group and more than 60 age group (*p*=0.1). The MB2 prevalence is also lower than in the over 60 age group than in the 40–59 age group but it is also not statistically significant (*p*=0.2).

The difference in prevalence is not statistically significant between males (*n*=50) and females (*n*=64) with *p*=0.5 in the 20–39 age group, between males (*n*=32) and females (*n*=34) with *p*=0.2 in the 40–59 age group, and between males (*n*=2) and females (*n*=0) in the 60 and more age group.

## 4. Discussion

The permanent first maxillary molar presents the greatest complexity and variation in the root canal system [8, 13]. Root canal morphologies can be analyzed in several ways including root canal staining [14], tooth clearing [15], and conventional and digital radiographs [16] that all have limitations.

CBCT imaging is considered to be useful in determining root canal morphology and can help endodontists to improve endodontic treatment outcomes [17]. Nowadays, a lot of MB2 population studies with CBCT exist [8, 17–23]. Our study presents another diagnostic feature for MB2 evaluation.

Though the MB2 canal ratio depends on factors such as sex and population [24], using different techniques and devices may affect the MB2 canal detection ratio [25]. Our CBCT study of 200 MB2 canals did not reveal any difference in prevalence with the 0.5 mm and 1 mm slice thicknesses; however, it revealed a statistically significant drop of MB2 prevalence with the 3 mm slice thickness, and no MB2 canals were visualized with the 10 mm slice thickness.

The same statistical fall was evident in gender and in every age group. Though some studies showed a higher MB2 prevalence in men than in women [8, 26–29], our study did not demonstrate a statistical difference in the MB2 prevalence between gender groups with the 3 mm slice thickness visualization.

Furthermore, it is considered that the decrease in visibility with CBCT for the higher age may be due to a decrease in the density of the cortical bone and a reduction in bone mass after 50 years of age [26, 30]. This study did not show any statistical difference among the age groups in total and the age groups according to gender.

The current study showed that the MB2 prevalence is also depend on the CBCT software settings without taking in account the race, age, and gender features.

## 5. Conclusion

Our study showed that evaluating the root canal system in first maxillary molars with 1 mm slice thickness is the best way to reduce inaccuracies caused by artifacts and attain the highest visualization of MB2 for both genders and every age group. Dentists should properly know how to use CBCT software and what settings to choose for proper root canal system evaluation.

## Data Availability

The data used to support the findings of this study are included within the article.

## References

[1] Schilder H. (1967). Filling root canals in three dimensions. *Dental Clinics of North America*.

[2] Schilder H. (1974). Cleaning and shaping the root canal. *Dental Clinics of North America*.

[3] Naseri M., Ahangari Z., Sharifi F., Sahebnasagh Z. (2015). Assessment of root morphology and apices of first and second maxillary molars in Tehran population. *Journal of Dental Materials and Techniques*.

[4] Siqueira J. F., Rôças I. N., Ricucci D., Hülsmann M. (2014). Causes and management of post-treatment apical periodontitis. *British Dental Journal*.

[5] Henry B. M. (1993). The fourth canal: its incidence in maxillary first molars. *Journal of the Canadian Dental Association*.

[6] Wong A. W., Zhu X., Zhang S., Li S. K., Zhang C., Chu C. H. (2015). Treatment time for non-surgical endodontic therapy with or without a magnifying loupe. *BMC Oral Health*.

[7] Haghanifar S., Moudi E., Madani Z., Farahbod F., Bijani A. (2017). Evaluation of the prevalence of complete isthmii in permanent teeth using cone-beam computed tomography. *Iranian Endodontic Journal*.

[8] Zheng Q.-h., Wang Y., Zhou X.-d., Wang Q., Zheng G.-n., Huang D.-m. (2010). A cone-beam computed tomography study of maxillary first permanent molar root and canal morphology in a Chinese population. *Journal of Endodontics*.

[9] Assadian H., Dabbaghi A., Gooran M. (2016). Accuracy of CBCT, digital radiography and cross-sectioning for the evaluation of mandibular incisor root canals. *Iranian Endodontic Journal*.

[10] Pour D., Arzi B., Shamshiri A. (2016). Assessment of slice thickness effect on visibility of inferior alveolar canal in cone beam computed tomography images. *Dental Research Journal*.

[11] Jasa G. R., Shimizu M., Okamura K. (2017). Effects of exposure parameters and slice thickness on detecting clear and unclear mandibular canals using cone beam CT. *Dentomaxillofacial Radiology*.

[12] Hekmatian E., Karbasi kheir M., Fathollahzade H., Sheikhi M. (2018). Detection of vertical root fractures using cone-beam computed tomography in the presence and absence of gutta-percha. *Scientific World Journal*.

[13] Badole G. P., Bahadure R. N., Warhadpande M. M., Kubde R. (2012). A rare root canal configuration of maxillary second molar: a case report. *Case Reports in Dentistry*.

[14] Neelakantan P., Subbarao C., Subbarao C. V. (2010). Comparative evaluation of modified canal staining and clearing technique, cone-beam computed tomography, peripheral quantitative computed tomography, spiral computed tomography, and plain and contrast medium-enhanced digital radiography in studying root canal morphology. *Journal of Endodontics*.

[15] Lee K.-W., Kim Y., Perinpanayagam H. (2014). Comparison of alternative image reformatting techniques in micro-computed tomography and tooth clearing for detailed canal morphology. *Journal of Endodontics*.

[16] Cohenca N., Simon J. H., Mathur A., Malfaz J. M. (2007). Clinical indications for digital imaging in dento-alveolar trauma. Part 2: root resorption. *Dental Traumatology*.

[17] Ratanajirasut R., Panichuttra A., Panmekiate S. (2018). A cone-beam computed tomographic study of root and canal morphology of maxillary first and second permanent molars in a Thai population. *Journal of Endodontics*.

[18] Kim Y., Lee S.-J., Woo J. (2012). Morphology of maxillary first and second molars analyzed by cone-beam computed tomography in a Korean population: variations in the number of roots and canals and the incidence of fusion. *Journal of Endodontics*.

[19] Calişkan M. K., Pehlivan Y., Sepetçioğlu F., Türkün M., Tuncer S. S. (1995). Root canal morphology of human permanent teeth in a Turkish population. *Journal of Endodontics*.

[20] Naseri M., Safi Y., Akbarzadeh Baghban A., Khayat A., Eftekhar L. (2016). Survey of anatomy and root canal morphology of maxillary first molars regarding age and gender in an Iranian population using cone-beam computed tomography. *Iranian Endodontic Journal*.

[21] Ghoncheh Z., Zade B. M., Kharazifard M. J. (2017). Root morphology of the maxillary first and second molars in an Iranian population using cone beam computed tomography. *Journal of Dentistry*.

[22] Silva E. J. N. L., Nejaim Y., Silva A. I. V., Haiter-Neto F., Zaia A. A., Cohenca N. (2014). Evaluation of root canal configuration of maxillary molars in a Brazilian population using cone-beam computed tomographic imaging: an *in vivo* study. *Journal of Endodontics*.

[23] Martins J. N. R., Marques D., Mata A., Caramês J. (2017). Root and root canal morphology of the permanent dentition in a Caucasian population: a cone-beam computed tomography study. *International Endodontic Journal*.

[24] Sert S., Bayirli G. (2004). Evaluation of the root canal configurations of the mandibular and maxillary permanent teeth by gender in the Turkish population. *Journal of Endodontics*.

[25] Shalabi R. M. A., Omer O. E., Glennon J., Jennings M., Claffey N. M. (2000). Root canal anatomy of maxillary first and second permanent molars. *International Endodontic Journal*.

[26] Betancourt P., Navarro P., Muñoz G., Fuentes R. (2016). Prevalence and location of the secondary mesiobuccal canal in 1,100 maxillary molars using cone beam computed tomography. *BMC Medical Imaging*.

[27] Fogel H. M., Peikoff M. D., Christie W. H. (1994). Canal configuration in the mesiobuccal root of the maxillary first molar: a clinical study. *Journal of Endodontics*.

[28] Betancourt P., Navarro P., Cantín M., Fuentes R. (2015). Cone-beam computed tomography study of prevalence and location of MB2 canal in the mesiobuccal root of the maxillary second molar. *International Journal of Clinical and Experimental Medicine*.

[29] Betancourt P., Fuentes R., Aracena Rojas S., Cantín M., Navarro Cáceres P. (2013). Prevalencia del segundo canal en la raíz mesiovestibular de los primeros molares maxilares mediante tomografía computarizada de haz de cono. *Avances en Odontoestomatología*.

[30] Hildebolt C. F. (1997). Osteoporosis and oral bone loss. *Dentomaxillofacial Radiology*.

